# CD4+ and CD8+ T cells and antibodies are associated with protection against Delta vaccine breakthrough infection: a nested case-control study within the PITCH study

**DOI:** 10.1128/mbio.01212-23

**Published:** 2023-09-01

**Authors:** Isabel Neale, Mohammad Ali, Barbara Kronsteiner, Stephanie Longet, Priyanka Abraham, Alexandra S. Deeks, Anthony Brown, Shona C. Moore, Lizzie Stafford, Susan L. Dobson, Megan Plowright, Thomas A. H. Newman, Mary Y. Wu, Crick COVID Immunity Pipeline, Edward J. Carr, Rupert Beale, Ashley D. Otter, Susan Hopkins, Victoria Hall, Adriana Tomic, Rebecca P. Payne, Eleanor Barnes, Alex Richter, Christopher J. A. Duncan, Lance Turtle, Thushan I. de Silva, Miles Carroll, Teresa Lambe, Paul Klenerman, Susanna Dunachie, On behalf of the PITCH Consortium

**Affiliations:** 1 Peter Medawar Building for Pathogen Research, Nuffield Department of Clinical Medicine, University of Oxford, Oxford, United Kingdom; 2 NDM Centre For Global Health Research, Nuffield Department of Clinical Medicine, University of Oxford, Oxford, United Kingdom; 3 Mahidol-Oxford Tropical Medicine Research Unit, Bangkok, Thailand; 4 Nuffield Department of Medicine, Pandemic Sciences Institute, University of Oxford, Oxford, United Kingdom; 5 Nuffield Department of Medicine, Wellcome Centre for Human Genetics, University of Oxford, Oxford, United Kingdom; 6 Oxford University Hospitals NHS Foundation Trust, Oxford, United Kingdom; 7 NIHR Health Protection Research Unit in Emerging and Zoonotic Infections, Institute of Infection, Veterinary and Ecological Sciences, University of Liverpool, Liverpool, United Kingdom; 8 Sheffield Teaching Hospitals NHS Foundation Trust, Sheffield, United Kingdom; 9 Department of Infection, Immunity and Cardiovascular Disease, University of Sheffield, Sheffield, United Kingdom; 10 Covid Surveillance Unit, The Francis Crick Institute, London, United Kingdom; 11 The Francis Crick Institute, London, United Kingdom; 12 UCL Department of Renal Medicine, Royal Free Hospital, London, United Kingdom; 13 UK Health Security Agency, Porton Down, United Kingdom; 14 UK Health Security Agency, London, United Kingdom; 15 National Emerging Infectious Diseases Laboratories, Boston University, Boston, Massachusetts, USA; 16 Department of Microbiology, Boston University School of Medicine, Boston, Massachusetts, USA; 17 Department of Biomedical Engineering, Boston University, Boston, Massachusetts, USA; 18 Department of Paediatrics, Oxford Vaccine Group, University of Oxford, Oxford, United Kingdom; 19 Translational and Clinical Research Institute Immunity and Inflammation Theme, Newcastle University, Newcastle, United Kingdom; 20 Translational Gastroenterology Unit, University of Oxford, Oxford, United Kingdom; 21 NIHR Oxford Biomedical Research Centre, University of Oxford, Oxford, United Kingdom; 22 Institute of Immunology and Immunotherapy, College of Medical and Dental Science, University of Birmingham, Birmingham, United Kingdom; 23 University Hospitals Birmingham NHS Foundation Trust, Birmingham, United Kingdom; 24 Department of Infection and Tropical Medicine, Newcastle upon Tyne Hospitals NHS Foundation Trust, Newcastle, United Kingdom; 25 Liverpool University Hospitals NHS Foundation Trust, Liverpool, United Kingdom; 26 Chinese Academy of Medical Science (CAMS) Oxford Institute (COI), University of Oxford, Oxford, United Kingdom; Max Planck Institute for Infection Biology, Berlin, Germany

**Keywords:** SARS-CoV-2, COVID-19, COVID vaccine, T cells, antibody, immunity, vaccine breakthrough, Delta

## Abstract

Serological correlates of protection against severe acute respiratory syndrome coronavirus 2 (SARS-CoV-2) infection after vaccination (“vaccine breakthrough”) have been described. However, T cell correlates of protection against breakthrough are incompletely defined, especially the specific contributions of CD4+ and CD8+ T cells. Here, 279 volunteers in the Protective Immunity from T Cells in Healthcare Workers (PITCH) UK cohort study were enrolled in a nested case-control study. Cases were those who tested SARS-CoV-2 PCR or lateral flow device (LFD) positive after two vaccine doses during the Delta-predominant era (*n* = 32), while controls were those who did not report a positive test or undergo anti-nucleocapsid immunoglobulin G (IgG) seroconversion during this period (*n* = 247). Previous SARS-CoV-2 infection prior to vaccination was associated with reduced odds of vaccine breakthrough. Using samples from 28 d after the second vaccine dose, before all breakthroughs occurred, we observed future cases had lower ancestral spike (S)- and receptor binding domain-specific IgG titers and S1- and S2-specific T cell interferon gamma (IFNγ) responses compared with controls, although these differences did not persist when individuals were stratified according to previous infection status before vaccination. In a subset of matched infection-naïve cases and controls, vaccine breakthrough cases had lower CD4+ and CD8+ IFNγ and tumor necrosis factor (TNF) responses to Delta S peptides compared with controls. For CD8+ responses, this difference appeared to be driven by reduced responses to Delta compared with ancestral peptides among cases; this reduced response to Delta peptides was not observed in controls. Our findings support a protective role for T cells against Delta breakthrough infection.

**IMPORTANCE:**

Defining correlates of protection against severe acute respiratory syndrome coronavirus 2 (SARS-CoV-2) vaccine breakthrough infection informs vaccine policy for booster doses and future vaccine designs. Existing studies demonstrate humoral correlates of protection, but the role of T cells in protection is still unclear. In this study, we explore antibody and T cell immune responses associated with protection against Delta variant vaccine breakthrough infection in a well-characterized cohort of UK Healthcare Workers (HCWs). We demonstrate evidence to support a role for CD4+ and CD8+ T cells as well as antibodies against Delta vaccine breakthrough infection. In addition, our results suggest a potential role for cross-reactive T cells in vaccine breakthrough.

## INTRODUCTION

Vaccination against severe acute respiratory syndrome coronavirus 2 (SARS-CoV-2) has been a crucial control strategy for the COVID-19 pandemic. Although new SARS-CoV-2 variants continue to emerge and cause infection, vaccine protection against severe COVID-19 remains high for otherwise healthy individuals (1, 2). Nonetheless, infection or re-infection after a two-dose primary vaccination course, so-called “vaccine breakthrough,” occurred during the emergence and spread of variants of concern (VOCs). As a result, booster (third) doses have been made available to all adults in many countries including the UK (3).

Understanding correlates of protection (CoP) against infection with SARS-CoV-2 could optimise delivery of current vaccines by informing boosting schedules and identifying individuals with suboptimal responses to vaccination. Identification of CoP could further facilitate licencing of modified or new vaccines based on immunogenicity and safety data rather than efficacy data (4), allowing new vaccines to more readily enter the marketplace.

Evidence supports a number of humoral CoP against infection with SARS-CoV-2. Neutralizing antibody (NAb) titers after vaccination correlate with vaccine efficacy and protection from infection in trials (4 – 7) and NAbs from prior infection with ancestral variant are associated with protection against infection with ancestral (8) and Alpha (9) variants. NAbs against SARS-CoV-2 are lower in the peri-infection period in those who experience Alpha vaccine breakthrough (10) and are lower at diagnosis in those who experience Delta vaccine breakthrough (11) compared with uninfected controls. Anti-ancestral spike (S) and receptor binding domain (RBD) immunoglobulin G (IgG) titers also correlate with protection from infection with the Alpha variant (4) and Delta variant (12 – 14) after vaccination. Ancestral variant RBD-specific memory B cell responses are lower in Delta breakthrough cases at the point of diagnosis compared to uninfected close contacts (15). Levels of serum anti-S and anti-RBD IgA 2–4 weeks after the second dose of BNT162b2 vaccine are also lower in those who experience subsequent Alpha or Gamma vaccine breakthrough compared to those who remain uninfected (16).

T cells play an important role in control of SARS-CoV-2 infection and in reducing disease severity upon infection (17, 18). In primary infection, strong SARS-CoV-2 specific CD4+ and CD8+ T cell responses are associated with reduced disease severity (19). Several studies have demonstrated that S-specific T cell responses are largely maintained against VOC (20 – 24), despite evasion of antibody responses (25 – 27). This supports T cells contributing to the continued effectiveness of vaccines against severe disease.

However, identifying a clear role for T cells in preventing infection is difficult due to the lack of sensitive, scalable T cell assays, and potential confounding effects of antibody responses (17, 28). Nonetheless, an increasing number of reports indicate that T cells are likely to be important in protection against SARS-CoV-2 infection. Unvaccinated individuals who are exposed to SARS-CoV-2 but remain antibody- and PCR-negative show T cell responses (29), and further studies demonstrate that pre-existing, cross-reactive T cell responses are associated with protection from overt infection (30, 31). In an unvaccinated cohort, seronegative individuals with detectable SARS-CoV-2-specific T cell responses had a lower risk of subsequent infection than seronegative individuals without positive T cell responses (32). More recently, higher whole-blood interferon gamma (IFNγ) responses to SARS-CoV-2 peptides among individuals who had received at least one vaccine dose were associated with a lower risk of breakthrough infection over the subsequent 6 mo period (33). There are also indications that the combination of cellular and humoral responses may be important in protecting against Delta and Omicron breakthrough infection. The combination of high NAb titer and high S1-specific IFNγ responses is associated with protection against breakthrough (34). Also, “high responders” to vaccination, characterized by high antibody, enriched CD4+ and CD8+ central memory 1, and durable CD8+ T cell responses, are more protected against symptomatic breakthrough compared to “low responders,” characterized by low antibody responses and more prevalent T effector memory 2 responses (35). Although these data indicate an important role for T cells, it is still unclear to what extent T cell protection is mediated by CD4+ versus CD8+ T cells, as in many studies only total T cell responses are investigated. However, depletion of vaccine-induced CD8+ T cells in rhesus macaques and subsequent challenge demonstrated that CD8+ T cells contribute to vaccine protection against SARS-CoV-2 replication in this model (36). Therefore, further research is needed to define possible T cell CoP against SARS-CoV-2 infection, in particular the contributions of CD4+ and CD8+ T cells to protection in humans.

Large-scale prospective studies with recruitment and sampling of individuals prior to breakthrough give the opportunity to better understand possible T cell CoP against SARS-CoV-2 (28). The Protective Immunity from T Cells in Healthcare Workers (PITCH) consortium is a prospective longitudinal cohort study of cellular immune responses to vaccination and infection in healthcare workers (HCWs) in the UK (http://www.pitch-study.org/), aligned with the wider SARS-CoV-2 immunity and reinfection evaluation (SIREN) study (37 – 39). The PITCH cohort demonstrated that an extended interval between the first and second dose of the BNT162b2 vaccine was associated with higher NAb and anti-S IgG binding titer and lower S-specific T cell response magnitude but higher interleukin-2 (IL-2) production compared to a shorter interval (22), findings which were also demonstrated in a separate study of older adults (40). The PITCH cohort includes individuals studied from April 2020 onwards, with data from pre-vaccination through to after the fourth vaccine dose, allowing sampling of well-characterized participants who subsequently experienced infection. The first vaccine breakthrough case in a PITCH participant was reported on the 10th of June 2021, at which time, Delta was the predominant variant circulating in the UK (41). A further 35 reports of vaccine breakthrough occurred in the cohort before the first Omicron cases were reported in the UK on the 27th of November 2021 (42). This offers an opportunity to use samples stored from before breakthrough infection to investigate CoP against infection after vaccination.

In the current study, we conducted a case-control study within the PITCH framework, using cases who reported infection during the Delta-predominant era in the UK and controls who did not experience vaccine breakthrough during this time. Cellular and humoral immunity at the timepoint 28 d after the second vaccine dose (V2 +28) was studied using an unmatched overall design in order to investigate whether peak post-vaccination responses are associated with subsequent breakthrough infection, irrespective of factors which may influence the level of response. A subset of cases and matched controls were selected for a more detailed analysis of cellular immunity. We observed lower S- and RBD-specific IgG binding titer and lower S1- and S2-specific T cell responses by IFNγ ELISpot assay in cases compared with controls at V2 +28. Using intracellular cytokine staining (ICS), we observed lower CD4+ and CD8+ IFNγ and tumor necrosis factor (TNF) responses to Delta peptides among cases compared with matched controls.

## RESULTS

### Breakthrough infections during the Delta-predominant era in the UK

Between the 10th of June and 27th of November 2021, there were 36 self-reports of vaccine breakthrough [positive polymerase chain reaction (PCR) or lateral flow device (LFD) test for SARS-CoV-2 >14 d after second vaccine dose and <7 d after third vaccine dose] in the PITCH cohort. These participants had been vaccinated with two doses of SARS-CoV-2 vaccine between 6th of January and 17th of July 2021. Twenty-six (72.2%) participants received BNT162b2 (Pfizer/BioNTech) and 10 (27.8%) received AZD1222 (Oxford/AstraZeneca) (Table 1). The median age of vaccine breakthrough cases was 43 [interquartile range (IQR) 31.5–49; range 22–72], 30 (83.3%) cases were female, and the majority (93.3%) of cases were of white ethnicity, reflecting the demographics of the underlying SIREN and PITCH study cohorts (22, 39). The median time between second vaccine dose and positive PCR or LFD test was 156 d (IQR 123.8–180.5; range 48–253).

**TABLE 1:** Demographic characteristics of vaccine breakthrough cases compared to controls and factors associated with vaccine breakthrough in multivariable logistic regression analysis

	All (*n* = 414)	Controls (*n* = 378)	Cases (*n* = 36)	OR^*a*^ (95% CI)	*P* value	Adjusted OR^*b*^ (95% CI)	*P* value
**Age**							
20–29	78 (18.8%)	70 (18.5%)	8 (22.2%)	1 (ref)	0.396	1 (ref)	0.456
30–39	86 (20.8%)	78 (20.6%)	8 (22.2%)	0.90 (0.31–2.56)		0.99 (0.34–2.86)	
40–49	109 (26.3%)	97 (25.7%)	12 (33.3%)	1.08 (0.43–2.89)		1.26 (0.49–3.44)	
50–59	97 (23.4%)	93 (24.6%)	4 (11.1%)	0.38 (0.10–1.24)		0.45 (0.11–1.51)	
60+	44 (10.6%)	40 (10.6%)	4 (11.1%)	0.88 (0.22–2.96)		1.19 (0.30–4.20)	
**Sex**							
Female	310 (74.9%)	280 (74.1%)	30 (83.3%)	1 (ref)	0.203	1 (ref)	0.078
Male	104 (25.1%)	98 (25.9%)	6 (16.7%)	0.57 (0.21–1.32)		0.46 (0.17–1.08)	
**Ethnicity (self-reported**)							
White	298 (84.4%)	270 (83.6%)	28 (93.3%)	1 (ref)	0.265	−	−
Asian	37 (10.5%)	36 (11.1%)	1 (3.3%)	0.27 (0.02–1.32)		−	
Other	18 (5.1%)	17 (5.3%)	1 (3.3%)	0.57 (0.03–2.93)		−	
Unreported	61	55	6	−		−	
**BMI (kg/m^2^**)							
Not obese (<30)	162 (89.5%)	150 (89.8%)	12 (85.7%)	1 (ref)	0.645	−	−
Obese (≥30.0)	19 (10.5%)	17 (10.2%)	2 (14.3%)	1.47 (0.22–6.01)		−	
Unreported	233	211	22	−		−	
**Vaccine type**							
BNT162b2	336 (81.2%)	310 (82.0%)	26 (72.2%)	1 (ref)	0.171	−	−
AZD1222	78 (18.8%)	68 (18.0%)	10 (27.8%)	1.75 (0.77–3.71)		−	
**Infection history**							
Naïve	244 (58.9%)	214 (56.6%)	30 (83.3%)	1 (ref)	0.001	1 (ref)	0.0009
Previous SARS-CoV-2	170 (41.1%)	164 (43.4%)	6 (16.7%)	0.26 (0.10–0.60)		0.25 (0.09–0.58)	

^*a*^ Crude odds ratio derived from single logistic regression.

^*b*^ Adjusted odds ratio derived from multiple logistic regression. The final model included and adjusted for age, sex, and infection history.

### Vaccine breakthrough is more common in individuals without history of previous infection

**Fig 1:**
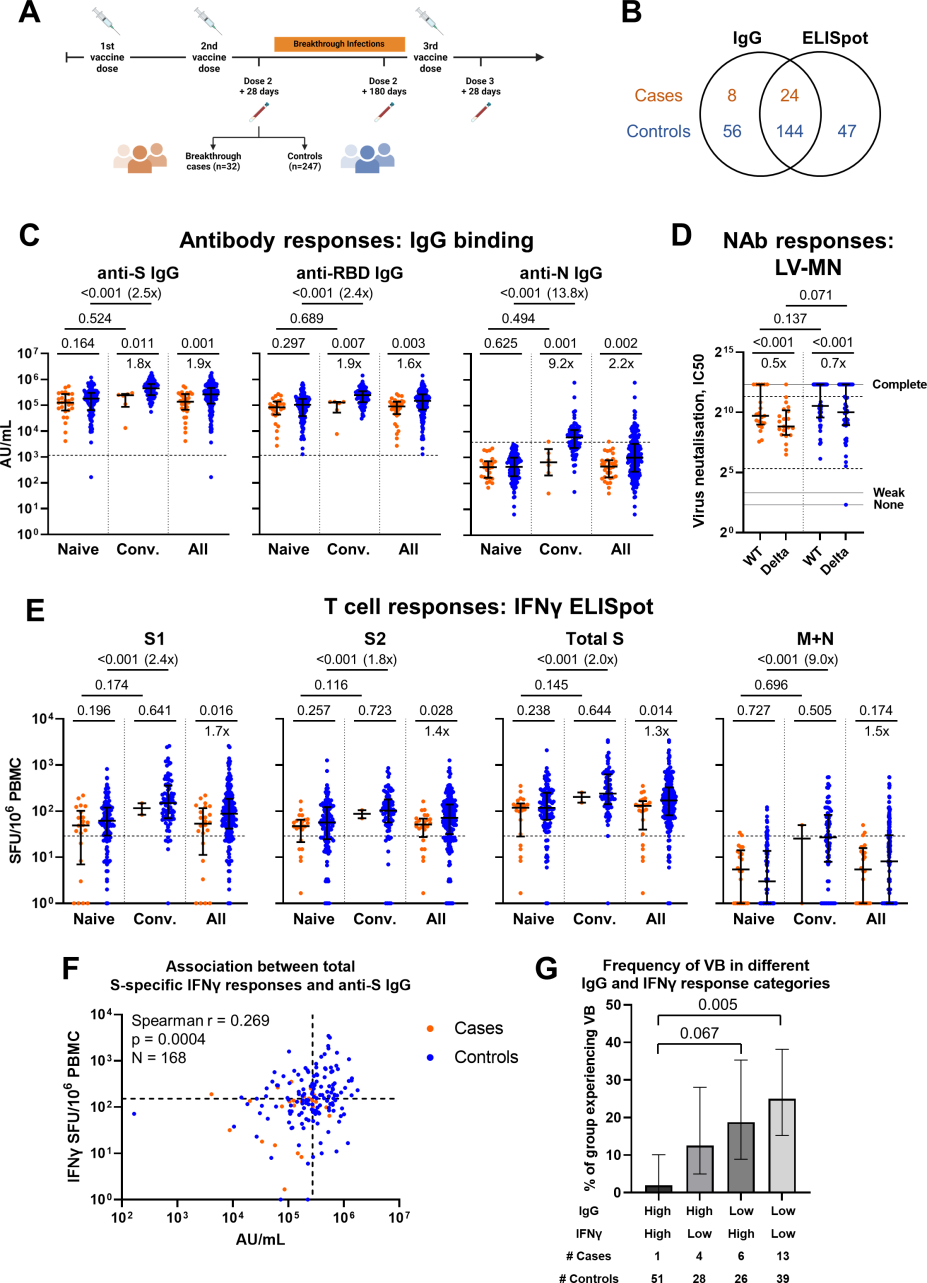
Study design and comparison of antibody and T cell responses between cases and controls at 28 d after the second vaccine dose. (**A**) Schematic representation of study design including vaccination and breakthrough infection time points. Created using Biorender.com. (**B**) Venn diagram illustrating number of subjects with IgG and/or IFNγ ELISpot measurements, by case/control status. (**C**) Ancestral SARS-CoV-2 spike- (S), receptor binding domain- (RBD) and nucleoprotein- (N) specific IgG binding titer in cases and controls according to infection history, as measured by MSD. Naïve represents those with no history of prior infection (cases *n* = 26, controls *n* = 123). Convalescent (Conv) represents those with a history of infection prior to vaccination (cases *n* = 6, controls *n* = 77). All represents summation of Naïve and Conv participants (cases *n* = 32, controls *n* = 200). Dashed lines represent threshold for a positive response (SARS-CoV-2 S IgG 1160 AU/mL, RDB IgG 1169 AU/mL, and N IgG 3874 AU/mL). (**D**) Neutralizing antibody titers against ancestral (WT) and Delta (B1.617.2) isolates in a subset of cases (*n* = 21) and controls (*n* = 41) measured by live virus microneutralization (LV-MN) assay. Area between IC50 = 40 and IC50 = 2,560 (dashed black lines) corresponds to the quantitative range of the assay. IC50 = 5, IC50 = 10, and IC50 = 5,120 (solid gray lines) represent no, weak, and complete inhibition, respectively. (**E**) T cell responses to peptide pools representing ancestral SARS-CoV-2 S1, S2, total S (summation of S1 and S2 responses) and membrane (M) and N responses in cases and controls according to infection history, as measured by IFNγ ELISpot assay. Naïve cases *n* = 22, controls *n* = 114. Conv cases *n* = 2, controls *n* = 77. All cases *n* = 24, controls *n* = 191. Dashed lines represent threshold for a positive response [29 SFU/10^6^ peripheral blood mononuclear cells (PBMC)] calculated as mean background (DMSO) response +2 standard deviations. (**F**) Correlation between anti-S IgG titers, as measured by MSD, and total S T cell responses, as measured by IFNγ ELISpot, for all cases (*n* = 24) and controls (*n* = 144) where both parameters were measured. Dashed lines represent the median value for each parameter. (**G**) Frequency of individuals with vaccine breakthrough (VB) in each quadrant of the graph in (**F**). Individuals are classified as having “High” or “Low” IgG and IFNγ responses on the basis of whether their response is above or below the median for each variable. Number of cases and controls per group are shown below the graph. Values of 0 are replaced with 1 for representation on the logarithmic scale. Orange circles represent cases, and blue circles represent controls. Bars represent median of each group (**C–E**). Error bars represent interquartile range (**C–E**) or 95% CI (**G**). Two-tailed *P* values derived from Mann-Whitney U tests [comparing cases to controls or naïve to convalescent, (**C–E**)], Wilcoxon matched pairs signed rank tests [comparing WT and Delta responses, (**D**)] or pairwise Fisher’s exact tests (**G**) shown above linking lines. For clarity, only adjusted *P* values < 0.1 are shown in (**G**). Fold change in median response shown below linking lines or in brackets, calculated as median response for controls divided by median response for cases, median response for convalescent divided by median response for naïve, or median response for Delta divided by median response for WT.

As expected, previous SARS-CoV-2 infection contributed to protection against further infection. Comparison of demographic characteristics of all breakthrough infection cases (*n* = 36) with controls, defined as eligible individuals in PITCH who did not report a positive PCR or LFD by, and were still enrolled at, the 27th of November 2021 or their third vaccine dose (*n* = 378), showed that the odds of vaccine breakthrough were higher among those previously infection-naïve compared to those previously infected with SARS-CoV-2 before vaccination (*P* = 0.0009, Table 1; Fig. S1). No significant association was found between odds of vaccine breakthrough infection and age, sex, body mass index (BMI), ethnicity, or vaccine type. All cases sampled at V2 +28 (*n* = 32) were then selected for a case-control study with controls (*n* = 247) being those who had been sampled at the V2 +28 timepoint and who did not report vaccine breakthrough or show evidence of anti-nucleocapsid (N) IgG seroconversion during the period of interest (Fig. 1A).

### Previous infection is an important determinant of IgG and IFNγ responses

We and others have previously shown that infection with SARS-CoV-2 prior to vaccination results in higher antibody and T cell responses to vaccination compared with those who are uninfected prior to vaccination (21, 22, 43 – 46). At the V2 +28 timepoint, among all individuals selected for our case-control study, naïve participants who were not infected before vaccination had lower anti-S, -RBD, and -N IgG binding titer and S1-, S2-, total S-, and membrane (M) and N-specific IFNγ ELISpot responses compared to convalescent individuals who had been infected before vaccination (Fig. S2, all *P* < 0.001). This again confirmed that previous infection was a strong determinant of both IgG and IFNγ responses to vaccination in this cohort.

### Cases have lower humoral and cellular responses than controls at V2+28

Comparing all cases with a V2 +28 sample to all available controls, cases had lower anti-S (*P* = 0.001), -RBD (*P* = 0.003), and -N (*P* = 0.002) IgG binding titer compared with controls (Fig. 1B and C). In a subset of cases (*n* = 21) and controls (*n* = 41), NAb titer against ancestral and Delta variants was measured (Fig. 1D). No difference in median NAb titer against ancestral variant was detected between cases and controls, although the sample size was small. Both cases and controls displayed a reduction in NAb titer to Delta compared to the ancestral virus (both *P* < 0.001). Cases also demonstrated lower ancestral S1- (*P* = 0.016), S2- (*P* = 0.028), and total S- (*P* = 0.014) specific IFNγ ELISpot responses compared to controls at the V2 +28 timepoint (Fig. 1E). A small number of naïve individuals (cases *n* = 2, controls *n* = 10) showed M and N responses above threshold, which could represent occult infection (29) or cross-reactivity to other endemic coronaviruses. For both IgG and IFNγ responses, when individuals were stratified according to previous infection status the difference between naïve cases and controls was no longer statistically significant (Fig. 1C and E). This could be in part due to the smaller sample sizes in these comparisons but may also be due to confounding in the overall analysis due to unmeasured aspects of the immune response to infection, such as mucosal responses. In a subset of individuals where cell availability permitted, IFNγ responses to peptide pools representing Delta S1 and S2 were assessed (Fig. S3). However, no difference was detected in responses between cases and controls, possibly due to the small sample size in this analysis.

There was a weak correlation between individual anti-S IgG titer and total S IFNγ response (Fig. 1F). Although previous infection played an important role in driving IgG and IFNγ responses, we were interested to investigate whether peak post-vaccination responses are associated with subsequent breakthrough infection, irrespective of factors that may influence the level of response. To investigate the combined effect of antibody and cellular responses on the risk of vaccine breakthrough, individuals were categorized as having “High” or “Low” anti-S IgG and S-specific IFNγ responses on the basis of whether the response was above or below the median, such that individuals were divided into four categories (Fig. 1G). The proportion of individuals experiencing vaccine breakthrough was significantly different between categories (Fisher’s exact test, *P* = 0.003). The proportion of those experiencing vaccine breakthrough was lowest among those with High IgG and High IFNγ responses, and significantly lower than those with Low IgG and Low IFNγ responses (pairwise Fisher’s exact test, adjusted *P* = 0.005). Those with either High IgG and Low IFNγ responses or Low IgG and High IFNγ responses had an intermediate risk of breakthrough; however, it cannot be concluded from this analysis whether high IgG or high IFNγ responses are individually associated with a lower rate of vaccine breakthrough infection.

### Naïve cases have lower S-specific T cell responses to Delta peptides compared to naïve controls at V2+28

**Fig 2:**
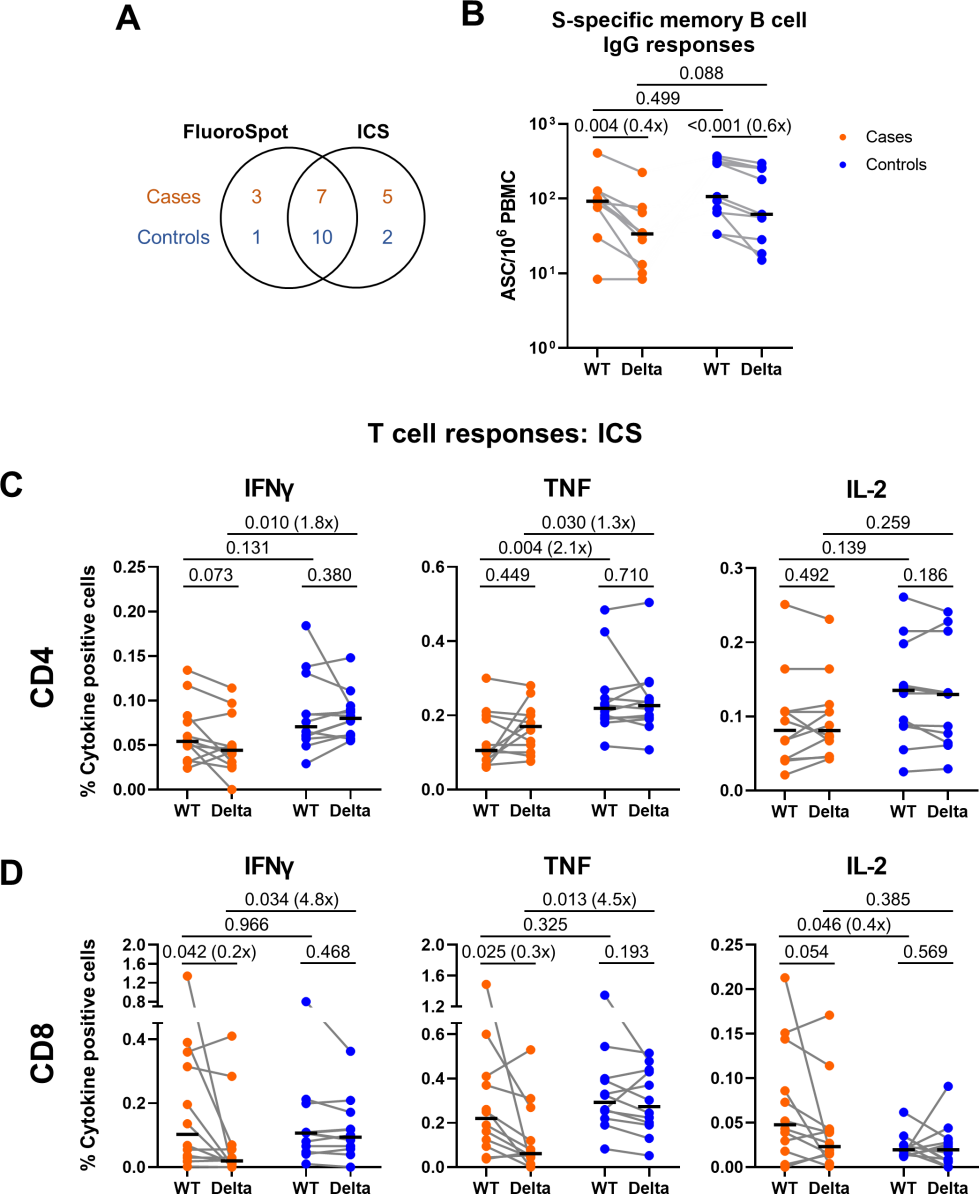
Comparison of memory B cell and T cell responses in a subset of cases and matched controls at 28 d after the second vaccine dose. (**A**) Venn diagram illustrating the overlapping subsets of cases and controls analyzed in (**B–D**). (**B**) Memory B cell responses against ancestral (WT) and Delta (B.1.617.2; AY.1, AY.2, AY.3) SARS-CoV-2 spike (S) in a subset of cases (*n* = 10) and matched controls (*n* = 11) measured by memory B cell IgG FluoroSpot. (**C**) Proportion of cells expressing IFNγ, TNF, or IL-2 in response to peptide pools representing ancestral (WT) or Delta (B.1.617.2) S in the CD4+ T cell population and (**D**) CD8+ T cell population, in a subset of cases and matched controls (*n* = 12 per group), as measured by intracellular cytokine staining and flow cytometry. Populations were analyzed by gating on single, live, CD3+ cells. Orange circles represent cases, and blue circles represent controls. Bars represent the median of each group. Paired data from the same individual are linked by gray lines. Two-tailed *P* values derived from Mann-Whitney U tests (comparing cases to controls) or Wilcoxon matched pairs signed rank tests (comparing WT and Delta responses) are shown above linking lines. Fold change in median response shown in brackets for comparisons where *P* < 0.05, calculated as median response for controls divided by median response for cases, or median response for Delta divided by median response for WT.

In a subset of cases and matched controls, all of whom were SARS-CoV-2 infection-naïve at vaccination, further analysis of cellular responses was undertaken. Ancestral and Delta S-specific memory B cell frequency was compared in a subset of cases (*n* = 10) and matched controls (*n* = 11) where peripheral blood mononuclear cell (PBMC) samples were available (Fig. 2A and B). There was no significant difference in ancestral or Delta S-specific memory B cell frequency among cases compared to controls (*P* = 0.499 and *P* = 0.088, respectively). However, there was high variation among controls and an outlier among cases, meaning that these data do not allow a conclusion to be drawn. There was a reduced response to Delta S compared to ancestral S among both cases (*P* = 0.004) and controls (*P* < 0.001).

CD4+ and CD8+ T cell responses were further investigated by ICS in a subset of cases and matched controls where PBMC were available (*n* = 12 per group). For CD4+ responses, there was a trend for a lower frequency of IFNγ and TNF expressing cells in cases compared with controls, for both ancestral and Delta S peptide pools (Fig. 2C). This difference was significant for TNF in response to ancestral S (*P* = 0.004) and IFNγ (*P* = 0.010) and TNF (*P* = 0.030) in response to Delta S peptides. There was high variability in the frequency of IL-2 expressing CD4+ T cells among cases and controls, with no difference detected in median frequency between groups. Across CD8+ T cell responses, there was a similar pattern, with a difference detected in frequency of IFNγ (*P* = 0.034) and TNF (*P* = 0.013) expressing cells in response to Delta S peptides (Fig. 2D). This difference appeared to be driven by a reduced response among cases to Delta peptides compared to ancestral peptides, which was not present in controls. CD8+ IL-2 responses appeared to be slightly higher in cases compared to controls in response to ancestral S (Fig. 2D, *P* = 0.046). However, overall CD8+ IL-2 responses were very low, and this trend appeared to be driven by one outlier with very high CD8+ responses across all cytokines.

Across IFNγ and TNF responses, on average over half of the response was contributed by CD4+ T cells, however, there was substantial variation between individuals (Fig. S4A and B), and it should be noted that for pragmatic purposes we used peptide pools of 18-mers, which are optimal for detection of CD4+ T cell responses and may therefore introduce bias against CD8+ responses in this analysis. A higher proportion of the TNF response to Delta peptide pools was contributed by CD4+ T cells among cases compared to controls (*P* = 0.028). CD4+ T cells contributed almost all of the IL-2 response to ancestral and Delta S peptide pools across the majority of cases and controls (Fig. S4C). Polyfunctionality analysis indicated that cases had a lower frequency of TNF+IFNγ+ double positive CD4+ and CD8+ T cells in response to Delta peptides compared with controls (Fig. S4E and G). There was also a trend for lower frequency of TNF+ single positive CD4+ and CD8+ T cells among cases compared to controls (Fig. S4D through G).

## DISCUSSION

This study examines immune responses associated with protection against SARS-CoV-2 breakthrough infection after two vaccine doses during the Delta-predominant era in the UK. Breakthrough infection was less likely in those with previous SARS-CoV-2 infection compared with those previously infection-naïve, consistent with other reports (47, 48). Cases showed lower anti-S and -RBD IgG binding titer as well as lower S1- and S2-specific IFNγ T cell responses by ELISpot assay, compared with controls, with previous infection being an important driver of these responses. The combination of high anti-S IgG responses and high S-specific IFNγ responses was associated with a lower frequency of breakthrough infection. In further analysis of infection-naïve individuals by ICS, lower CD4+ and CD8+ IFNγ and TNF responses to Delta S peptide pools were found among cases compared to controls. Our detailed look at S-specific T cell function with sensitive assays provides further evidence of a role for T cells in protection to add to established serological CoP from large studies of humoral function.

The scale of the PITCH consortium provides sufficient power to detect important differences between groups (21, 22). To make use of this large dataset, an unmatched overall design was therefore chosen, combining naïve and previously infected individuals for the main analysis to determine whether the level of humoral and cellular responses predict subsequent vaccine breakthrough irrespective of factors that may influence the level of response. There may, therefore, be some confounding in this analysis due to previous infection, for example, if previous infection is a marker of mucosal responses, not measured here. Nonetheless, nonmechanistic associations are still useful.

Our finding that anti-S and -RBD IgG binding titers differ between those who go on to experience breakthrough and those who do not experience overt infection is consistent with a number of other reports that previously demonstrated an association between these parameters and risk of breakthrough infection (4, 12, 14). The SIREN study is also well placed to look at serological CoP against reinfection in a large cohort of HCWs that includes several cases and controls from this study. SIREN recently reported that anti-S antibody levels and NAb titers correlated with protection against reinfection during the Alpha wave, prior to vaccination (9).

In this study, vaccine breakthrough was associated with lower IFNγ T cell responses by ELISpot, and lower CD4+ and CD8+ IFNγ and TNF responses to Delta peptides in ICS, even using a modest sample size. There are increasing reports indicating an important role for T cell CoP alongside serological CoP (32 – 34). Our findings using the IFNγ ELISpot assay help to validate these reports. Our more detailed findings using ICS support a role for both CD4+ and CD8+ T cells in protecting against susceptibility to overt infection with the Delta variant. The apparent drop in CD8+ IFNγ and TNF responses to Delta peptides compared with ancestral peptides among cases was particularly striking and suggests a role for cross-reactive CD8+ T cells in protection against infection with the Delta variant. Previous studies in a rhesus macaque model support a role for CD8+ T cell responses in protection against re-challenge in the context of waning antibody levels (49) and a direct role in vaccine-induced protection against SARS-CoV-2 replication (36). In addition, infection-naïve vaccinees with high antibody responses have enriched CD4+ and CD8+ central memory 1 cell responses and durable CD8+ T cell responses to ancestral and VOC S peptides compared to those with low antibody responses and are protected against symptomatic breakthrough infection with Delta or Omicron (35). Virus-specific CD8+ T cells are associated with protection against infection with other viral pathogens. For example, in the context of influenza A virus (IAV) epidemics, each fold increase in pre-existing H3N2- or seasonal H1N1-specific CD8+ T cell response is associated with reduced odds of infection with H3N2 or pandemic H1N1 IAV, respectively (50). IFNγ+IL-2- CD8+ T cells specific for conserved IAV core protein epitopes are also associated with protection against symptomatic disease caused by pandemic H1N1 IAV (51). Several human studies investigating immune responses around the time of SARS-CoV-2 breakthrough infection have not identified differences in T cell responses between cases and controls but have identified differences in terms of NAb titer (52, 53) and memory B cell responses (15). The difference in results between these studies and our study may be due to different timing of sampling and/or SARS-CoV-2 variant.

Our study focused on the Delta wave of infections in the UK, during a time at which the study participants had received two vaccine doses. However, the Delta variant has been largely displaced by Omicron in the UK and globally, and in the UK a third SARS-CoV-2 vaccine dose has been recommended to all adults (3) with further doses for those at increased risk and HCWs. The generalisability of the findings of this study to Omicron breakthroughs occurring after two or three vaccine doses needs further evaluation. Studies of CoP against Omicron have reported mixed results. A study investigating breakthrough after two vaccine doses found that there was no difference in anti-RBD titer between cases who subsequently experienced Omicron breakthrough and controls (13). Other studies did not identify differences in anti-S IgG titer (54), anti-RBD IgG titer, Omicron NAb titer, or T cell IFNγ responses (55) after a third vaccine dose between those who subsequently experienced Omicron breakthrough and those who did not. However, others have reported that low anti-S antibody titer and weak neutralization post-third dose are associated with a higher risk of breakthrough (56). In the peri-infection period, low pre-infection anti-S IgG titer is associated with breakthrough (57); however, it has also been shown that Omicron breakthrough can occur in those with high NAb titers (58).

### Limitations

Important limitations are as follows. (i) The sample size for cases is relatively small, despite follow-up of a large number of individuals in the overall PITCH consortium, because breakthrough infection during this time (pre-Omicron) was still relatively rare. We used the maximum sample size available for this case-control study for each assay type; however, the small number of cases means there was inadequate power to conduct a fully adjusted analysis of the effect of humoral and cellular responses on odds of vaccine breakthrough using logistic regression. The small sample size may also limit the generalisability of the findings. There are unfortunately no further stored PBMC samples available for analysis. (ii) Samples were not obtained during infection from which to determine sequence information, so we were unable to confirm whether these breakthrough infections were attributable to the Delta variant. However, during the period in which the infections occurred, Delta was the predominant variant in the UK, causing ≥89.9% of genotyped infections throughout this time (41). This approach using the timing of infection to indicate which variant is likely the cause of infection is in line with approaches used in other studies (1). (iii) Our study was not designed to explore specific co-morbidities, particularly immunosuppression, as risk factors for breakthrough infection (59, 60). This is addressed in the subsequent VIBRANT study (https://vibrant-research-study.org/). (iv) All observational studies carry the risk of bias. Adherence to LFD and PCR testing recommendations was not monitored within PITCH, thus there may be some mis-classification of controls. Although serology from later follow-up points was used to confirm as far as possible that controls were not infected during the period of interest, up to 60% of vaccinated individuals may not undergo anti-N seroconversion upon infection (61). It is likely there were differences in exposure to SARS-CoV-2 between individuals dependent on their exact HCW role, for example, seeing more than one COVID-19 patient per day has previously been associated with a higher risk of infection among HCWs (4); however, this information was not available for us to take in to account in analysis. (v) We did not investigate mucosal immunity in this study, as mucosal samples were not obtained from PITCH participants at that time. Mucosal immune responses do not necessarily correlate with responses in the peripheral circulation (62) and may play an important role in protection against SARS-CoV-2 infection, particularly in individuals with hybrid immunity (63). Ongoing PITCH research now incorporates measurement of mucosal immunity. Nonetheless, this study has several strengths, primarily that the PITCH cohort is very well characterized, and the prospective nature of the study allowed investigation at a pre-specified timepoint reflecting peak vaccine responses, prior to infection.

Questions remain regarding the mechanism of action of T cells in protection against breakthrough infection. CD8+ T cells may contribute to the control of replication after infection, as demonstrated in a rhesus macaque model (36). CD4+ T cells are important for providing T cell help to support CD8+ T cells and for generation of antibody responses. There may also be a role for HLA type and which epitopes are immunodominant in different individuals. For example, it has been shown that there is an association between the HLA-DQB1*06 allele and higher anti-RBD IgG titer after vaccination with AZD1222, and that this may play a role in protection from breakthrough infection with ancestral and Alpha strains (64).

Overall, our study supports a role for T cell responses alongside antibody responses in protection against detectable infection during the period after double vaccination, over which antibody responses have been shown to wane (21, 65, 66). Moreover, our results suggest a potential role for cross-reactive CD8+ T cell responses. Development of sensitive, quantitative T cell assays that can be used at scale in large cohorts in a range of settings will allow further integrated study of humoral and cellular adaptive immunity and inform vaccine surveillance and effectiveness monitoring.

## MATERIALS AND METHODS

### Study design and sample collection

The PITCH consortium has been described previously (21, 22, 43). Briefly, participants in this prospective, observational, cohort study were recruited from five university hospitals in England (Birmingham, Liverpool, Newcastle, Oxford, and Sheffield). Recruitment of consenting individuals was by word of mouth, hospital e-mail communications, and from hospital-based staff screening programs for SARS-CoV-2, including HCWs enrolled in the national SIREN study at three sites (Liverpool, Newcastle, and Sheffield). Eligible participants were adults aged 18 or over, currently working as HCWs, including allied support and laboratory staff, or were volunteers linked to the hospital. Individuals were defined as SARS-CoV-2 naive or previously-infected based on documented PCR and/or serology results from local NHS trusts, or the Meso Scale Discovery (MSD) assay S and N antibody results if these data were not available locally, as described previously (22).

PITCH is a sub-study of the SIREN study, which was approved by the Berkshire Research Ethics Committee, Health Research 250 Authority (IRAS ID 284460, REC reference 20/SC/0230), with PITCH recognized as a sub-study on 2 December 2020. SIREN is registered with ISRCTN (Trial ID: 252 ISRCTN11041050). Some participants were recruited under aligned study protocols. In Birmingham, participants were recruited under the “Determining the immune response to SARS-CoV-2 infection in convalescent health care workers” (COCO) study (IRAS ID: 282525). In Liverpool, some participants were recruited under the “Human immune responses to acute virus infections” Study (16/NW/0170), approved by North West - Liverpool Central Research Ethics Committee on 8 March 2016 and amended on 14 September 2020 and 4 May 2021. In Oxford, participants were recruited under the GI Biobank Study 16/YH/0247, approved by the research ethics committee (REC) at Yorkshire and the Humber - Sheffield Research Ethics Committee on 29 July 2016, which has been amended for this purpose on 8 June 2020. In Sheffield, participants were recruited under the Observational Biobanking study STHObs (18/YH/0441), which was amended for this study on 10 September 2020. The study was conducted in compliance with all relevant ethical regulations for work with human participants, and according to the principles of the Declaration of Helsinki (2008) and the International Conference on Harmonization (ICH) Good Clinical Practice (GCP) guidelines. Written informed consent was obtained for all participants enrolled in the study.

The current study is nested within PITCH. A vaccine breakthrough case was defined as a self-reported positive PCR or LFD test for SARS-CoV-2 >14 d after the second vaccine dose and <7 d after the third vaccine dose. The period of interest for cases was when Delta was the predominant circulating SARS-CoV-2 variant in the UK, defined here as the 6th of June to the 27th of November 2021, with the 27th of November taken as the cut-off date as this was the date of the first reported cases of SARS-CoV-2 Omicron variant in the UK (42). Throughout this period, ≥89.9% of genotyped infections across England were attributable to the Delta variant (41). During this period, all UK HCWs were requested to undertake twice-weekly asymptomatic testing for SARS-CoV-2 using self-swab LFDs that were provided free of charge by workplaces, pharmacies, and by postal delivery from the government website. Symptomatic PCR testing was readily available at this time free of charge from hospital trusts and UK community testing programs. Adherence to this testing was not measured within PITCH; however, PITCH participants are asked about SARS-CoV-2 infections at every clinic visit.

The current study has a case-control design (Fig. S1). Eligibility criteria were enrolment in PITCH by the 30th of June 2021 and being double vaccinated by the 31st of July 2021. For correlates analysis, all those meeting the criteria of a vaccine breakthrough case and who had been sampled at the V2 +28 timepoint were included. Controls were those who had been sampled at the V2 +28 timepoint and did not report a positive PCR or LFD by the 27th of November 2021 or 7 d after the third vaccine dose (V3 +7), whichever was earlier, and who did not show detectable N seroconversion (defined as anti-N IgG titer over the positivity threshold previously defined using pre-pandemic samples (43), and at least double the individual’s baseline value) in available follow-up data during this time (Table S1 and S2).

A subset of cases was selected on the basis of sample availability for further analysis using ICS and memory B cell FluoroSpot. Controls were matched with these cases for each assay, with matching based on age, sex, previous infection status at enrolment, vaccine manufacturer, and dose interval (Table S3). Cases in this matched analysis had been enrolled at multiple sites, whereas controls had all been enrolled at the Oxford site. All experiments for this subset analysis were conducted at the Oxford site.

Participants were sampled for the current study between 4th of January 2021 and 13th of August 2021, approximately 28 d after their second vaccine dose (median 28 d, IQR 26–33 d). PBMC, plasma, and serum were separated and cryopreserved. The majority of participants had been investigated for previous reports of the PITCH cohort (21 – 23, 43).

### Demographic analysis

Demographic characteristics of vaccine breakthrough cases compared to the PITCH cohort were initially analyzed by univariable logistic regression to estimate the crude odds ratio for vaccine breakthrough for each variable. A likelihood ratio test was conducted with the null hypothesis of no association between vaccine breakthrough and each variable. An adjusted model was derived by backward stepwise regression, with age and sex included as *a priori* confounders. Variables were removed from the model sequentially, beginning with those with the least effect in univariable regression. A likelihood ratio test was conducted after each removal, and only those variables with evidence of association with vaccine breakthrough (*P* < 0.05) were retained in the model. Analysis was performed using R version 4.1.2 (the R Foundation for Statistical Computing, Vienna, Austria, 2021. URL https://www.R-project.org/). Confidence intervals were derived using the confint package.

### T cell IFNγ ELISpot assay

IFNγ ELISpot assays were performed using the Human IFNγ ELISpot Basic kit (Mabtech 3420–2A), according to the PITCH ELISpot Standard Operating Procedure as previously published (43). Cryopreserved PBMC were thawed in R10 media [RPMI 1640 with 10% (vol/vol) Fetal Bovine Serum, 2 mM L-Glutamine, and 1 mM Penicillin/Streptomycin (all Sigma)] and rested for 3–6 h in a humidified incubator at 37°C, 5% CO_2_. MultiScreen-IP filter plates (Millipore) were coated with 10 µg/mL capture antibody (clone 1-D1K, Mabtech) for 3–8 h and blocked with R10 or Rab10 [RPMI 1640 with 2 mM L-Glutamine and 1 mM Penicillin/Streptomycin supplemented with 10% (vol/vol) Human AB Serum (Sigma)] for 1–8 h at room temperature (RT). PBMC were resuspended in R10 or Rab10, plated in triplicate at 2 × 10^5^ cells/well, and stimulated as follows. PBMC were stimulated with overlapping peptide pools (18-mers with 10 amino acid overlap, Mimotopes) spanning the S or M and N SARS-CoV-2 proteins at a final concentration of 2 µg/mL for 16–18 h in a humidified incubator at 37°C, 5% CO_2_. For selected individuals, pools spanning the S protein of the Delta variant were included. Pools consisting of CMV, EBV, and influenza peptides at a final concentration of 2 µg/mL (CEF; Proimmune) and concanavalin A or phytohemagglutinin L (PHA-L, Sigma) were used as positive controls. DMSO was used as the negative control at an equivalent concentration to the peptides. After the incubation period and all subsequent steps plates were washed with PBS supplemented with 0.05% (vol/vol) Tween20 (Sigma). Detection antibody (clone 7-B6-1, Mabtech) was added and incubated for 2–4 h at RT. Plates were washed and streptavidin-ALP (Mabtech) was added for 1–2 h at RT, before washing again and addition of 1-step NBT/BCIP substrate solution (Thermo Scientific) for 5–7 min at RT. Color development was stopped by washing with tap water. Air-dried plates were scanned and analyzed with the AID Classic ELISpot Reader (software version 8.0, Autoimmune Diagnostika GmbH, Germany) or ImmunoSpot S6 Alfa Analyser (Cellular Technology Limited LLC, Germany). Antigen-specific responses were calculated by subtracting the mean number of spots in the DMSO wells from the test wells and expressed as spot-forming units (SFU)/10^6^ PBMC. Samples with a mean spot value >50 in the DMSO wells were excluded from the analysis.

### MSD IgG binding assay

IgG responses to SARS-CoV-2, SARS-CoV-1, MERS-CoV, and seasonal coronaviruses were measured using a multiplexed MSD immunoassay (The V-PLEX COVID-19 Coronavirus Panel 3 (IgG) Kit (cat. no. K15399U) from Meso Scale Discovery, Rockville, MD USA), as reported previously (21, 22). A MULTI-SPOT 96-well, 10 spot plate was coated with three SARS CoV-2 antigens (S, RBD, N), SARS-CoV-1 and MERS-CoV spike trimers, spike proteins from seasonal human coronaviruses, HCoV-OC43, HCoV-HKU1, HCoV-229E and HCoV-NL63, and bovine serum albumin (negative control). Antigens were spotted at 200–400 µg/mL (MSD Coronavirus Plate 3). Multiplex MSD assays were performed as per the manufacturer’s instructions. To measure IgG antibodies, 96-well plates were blocked with MSD Blocker A for 30 min. Following washing with washing buffer, serum or plasma samples diluted 1:1,000–30,000 in diluent buffer, MSD standard, and undiluted internal MSD controls were added to the wells. After 2 h incubation and washing, detection antibody (MSD SULFO-TAG anti-human IgG antibody, 1/200) was added. Following washing, MSD GOLD read buffer B was added and plates were read using a MESO SECTOR S 600 reader. The standard curve was established by fitting the signals from the reference standard using a 4-parameter logistic model. Concentrations of samples were determined from the electrochemiluminescence signals by back-fitting to the standard curve and multiplying by the dilution factor. Concentrations are expressed in Arbitrary Units/mL (AU/mL). Thresholds were previously determined for each SARS-CoV-2 antigen based on the mean concentrations measured in 103 pre-pandemic sera +3 Standard Deviations (43). Thresholds were as follows: S, 1160 AU/mL; RBD, 1169 AU/mL; and N, 3874 AU/mL.

### Memory B cell IgG FluoroSpot assay

In a subset of cases and matched controls for which PBMC were available, B cell memory responses were characterized by IgG FluoroSpot assay, according to methods previously reported (21). Cryopreserved PBMC were thawed and rested for 4–6 h in R10 media, then cultured in R10 supplemented with 2 mM Sodium Pyruvate, 2 mM MEM Non-Essential Amino Acids, 200 nM 2-Mercaptoethanol (all Life Technologies), 1 µg/mL R848, and 10 ng/mL IL-2 (Mabtech Human memory B cell stimpack) for 68–72 h in a humidified incubator at 37°C, 5% CO_2_, for polyclonal stimulation. Using the Human IgA/IgG FluoroSpotFLEX Kit (Mabtech), stimulated PBMC were then added at 2 × 10^5^ cells/well to FluoroSpot plates coated with 10 µg/mL ancestral and Delta SARS-CoV-2 S glycoprotein, PBS (negative control), or capture mAbs (positive control). Plates were incubated for 18 h in a humidified incubator at 37°C, 5% CO_2_ and developed according to the manufacturer’s instructions (Mabtech). Analysis was carried out with AID ELISpot software 8.0 (Autoimmun Diagnostika). Samples were tested in duplicate or triplicate where cell counts permitted. Antigen-specific responses were calculated by subtracting the mean number of spots in the PBS wells from the test wells and expressed as antibody-secreting cells (ASC)/10^6^ PBMC.

### Intracellular cytokine staining assay

In a subset of cases and matched controls for which PBMC were available, T cell responses were characterized further using ICS after stimulation with overlapping SARS-CoV-2 peptide pools, according to methods reported previously (21). Cryopreserved PBMC were thawed and rested in R10 media for 4–6 h then plated at 1 × 10^6^ cells/well in a 96-well U-bottom plate together with co-stimulatory molecules anti-CD28 and anti-CD49d (both BD) at 1 µg/mL final concentration. Peptide pools spanning ancestral and Delta S proteins were added at 2 µg/mL final concentration for each peptide pool. DMSO (Sigma) was used as the negative control at the equivalent concentration to the peptides. As a positive control, cells were stimulated with 1× cell activation cocktail containing phorbol-12-myristate 13-acetate (PMA) at 81 µM and ionomycin at 1.3 µM final concentration (Biolegend). Cells were then incubated in a humidified incubator at 37°C, 5% CO_2_ for 1 h before incubating for a further 15 h in the presence of 5 µg/mL Brefeldin A (Biolegend). Flow cytometry staining was then performed as follows. Cell staining buffer (Biolegend) was used for staining and washing before fixation, with 1× Perm/Wash buffer (BD) used after fixation. At the end of the culture period, PBMC were washed once and subsequently stained with near-infrared fixable live/dead stain (Invitrogen), fluorochrome-conjugated primary human-specific antibodies against CD4, CD8, and CD14 (all Biolegend) and human Fc blocking reagent (Miltenyi Biotec) for 20 min at 4°C in the dark. PBMC were washed and then fixed and permeabilized in Cytofix/Cytoperm buffer (BD) for 20 min at 4°C in the dark. PBMC were then washed followed by staining with primary human-specific antibodies against CD3, IFNγ, TNF (all Biolegend), IL-2 (eBioscience), and human Fc blocking reagent for 20 min at 4°C in the dark. PBMC were washed, resuspended in cell staining buffer, and stored at 4°C for up to 6 h until acquisition. Samples were acquired on a MACSQuant Analyzer 10 and X (Miltenyi Biotec), and analysis was performed using FlowJo software version 10.8.1 (BD Biosciences). An example gating strategy is shown in Fig. S5. Details of antibodies used are listed in Table S4.

### High-throughput live virus microneutralization assay

In a subset of cases and controls, high-throughput live virus microneutralization assays were performed as described previously (67, 68). Briefly, Vero E6 cells (Institute Pasteur) (69) at 90–100% confluency in 384-well format were first titrated with varying MOIs of each SARS-CoV-2 variant and varying concentrations of a control monoclonal nanobody in order to normalize for possible replicative differences between variants and select conditions equivalent to wild-type virus. Following this calibration, cells were infected in the presence of serial dilutions of patient serum samples. 24 h after infection, cells were fixed with 4% final Formaldehyde, permeabilized with 0.2% TritonX-100, 3% BSA in PBS (vol/vol), and stained for SARS-CoV-2 N protein using Biotin-labeled-CR3009 antibody produced in-house in conjunction with Alexa488-Streptavidin (Invitrogen S32354) and cellular DNA using DAPI (70). Whole-well imaging at 5× was carried out using an Opera Phenix (Perkin Elmer), and fluorescent areas and intensity were calculated using the Phenix-associated software Harmony (Perkin Elmer). Inhibition was estimated from the measured area of infected cells/total area occupied by all cells. The inhibitory profile of each serum sample was estimated by fitting a 4-parameter dose response curve executed in SciPy. Neutralizing antibody titers are reported as the fold-dilution of serum required to inhibit 50% of viral replication (IC50) and are further annotated if they lie above the quantitative (complete inhibition) range (titer >2,560), below the quantitative range (<40) but still within the qualitative range (i.e., partial inhibition is observed but a dose-response curve cannot be fit because it does not sufficiently span the IC50) or if they show no inhibition at all. For plotting, complete, weak, and no inhibition were assigned 5,120, 10, and 5, respectively. Details of viruses used in the study are listed in Table S5.

### Statistical analysis

Continuous variables are displayed as the median and interquartile range. Unpaired comparisons between the two groups were analyzed using the Mann-Whitney U test. Paired comparisons between groups were analyzed by the Wilcoxon matched-pairs signed-rank test. Comparison between four groups was carried out using Fisher’s exact test and post-hoc paired Fisher’s exact tests adjusted with the Bonferroni correction. *P* values are two-tailed. Statistical analyses were performed using R version 4.1.2 (The R Foundation for Statistical Computing, Vienna, Austria, 2021. URL https://www.R-project.org/) and GraphPad Prism 9.4.0.

## SUPPLEMENTAL MATERIAL

Figure S1 mbio.01212-23-s0001.docx Flow chart of study design.

Figure S2 mbio.01212-23-s0002.docx Additional antibody and T cell data.

Figure S3 mbio.01212-23-s0003.docx Additional T cell data.

Figure S4 mbio.01212-23-s0004.docx Additional flow cytometry data.

Figure S5 mbio.01212-23-s0005.docx Flow cytometry gating strategy.

Secondary Author List mbio.01212-23-s0006.docx List of consortium authors.

Supplemental Tables mbio.01212-23-s0007.docx Tables S1-S5.

ASM does not own the copyrights to Supplemental Material that may be linked to, or accessed through, an article. The authors have granted ASM a non-exclusive, world-wide license to publish the Supplemental Material files. Please contact the corresponding author directly for reuse.
