# Supplementary material for: CD4+ and CD8+ T cells and antibodies are associated with protection against Delta vaccine breakthrough infection: a nested case-control study within the PITCH study
Source: mBio. 2023 Sep 1;14(5):e01212-23. doi: 10.1128/mbio.01212-23 (PMC10653804; doi:10.1128/mbio.01212-23)
Supplement: Figure S1 — Flow chart of study design. [file mbio.01212-23-s0001.docx]

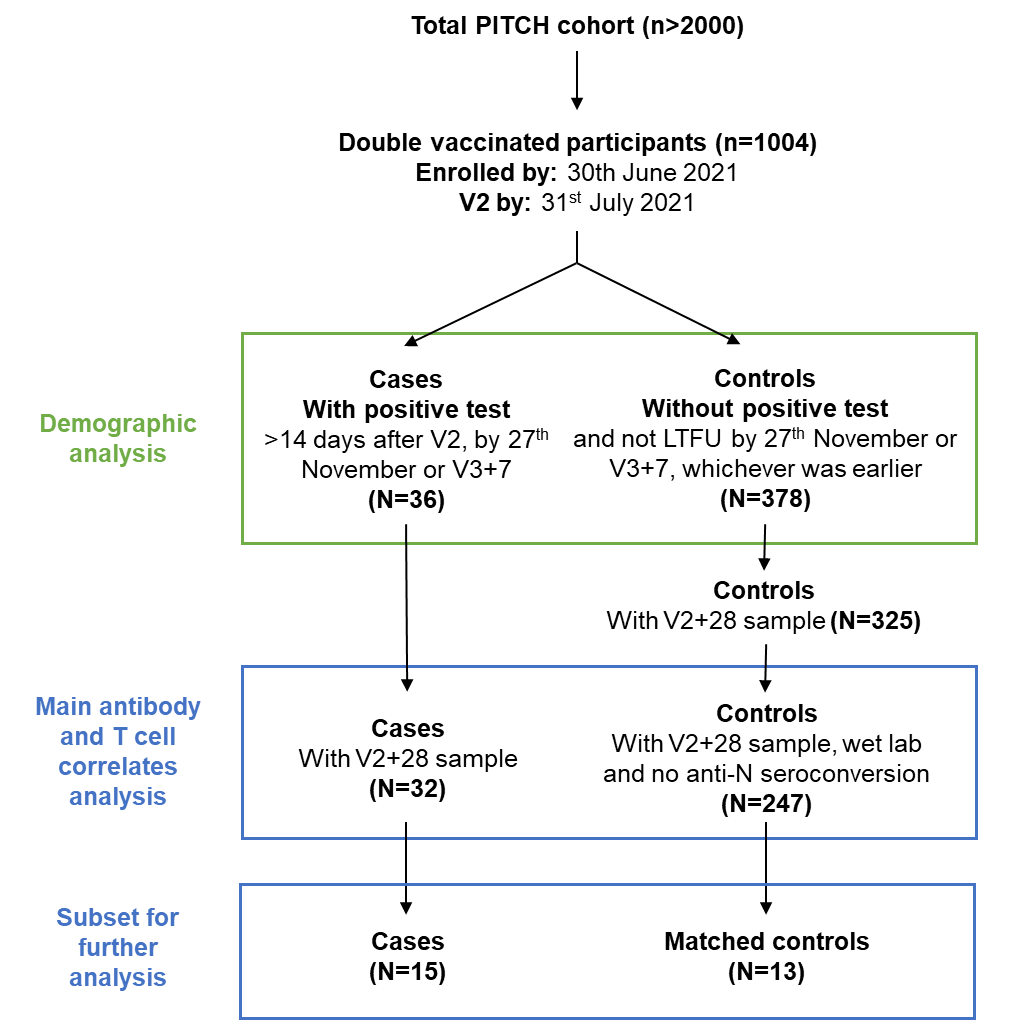


**Figure S1. Flow chart indicating overall design of this study within the PITCH cohort. V2 refers to second vaccination.** V2+28 refers to 28 days after the second vaccine dose. V3+7 refers to 7 days after the third vaccine dose. LTFU refers to lost to follow-up.
