# Supplementary material for: CD4+ and CD8+ T cells and antibodies are associated with protection against Delta vaccine breakthrough infection: a nested case-control study within the PITCH study
Source: mBio. 2023 Sep 1;14(5):e01212-23. doi: 10.1128/mbio.01212-23 (PMC10653804; doi:10.1128/mbio.01212-23)
Supplement: Figure S2 — Additional antibody and T cell data. [file mbio.01212-23-s0002.docx]

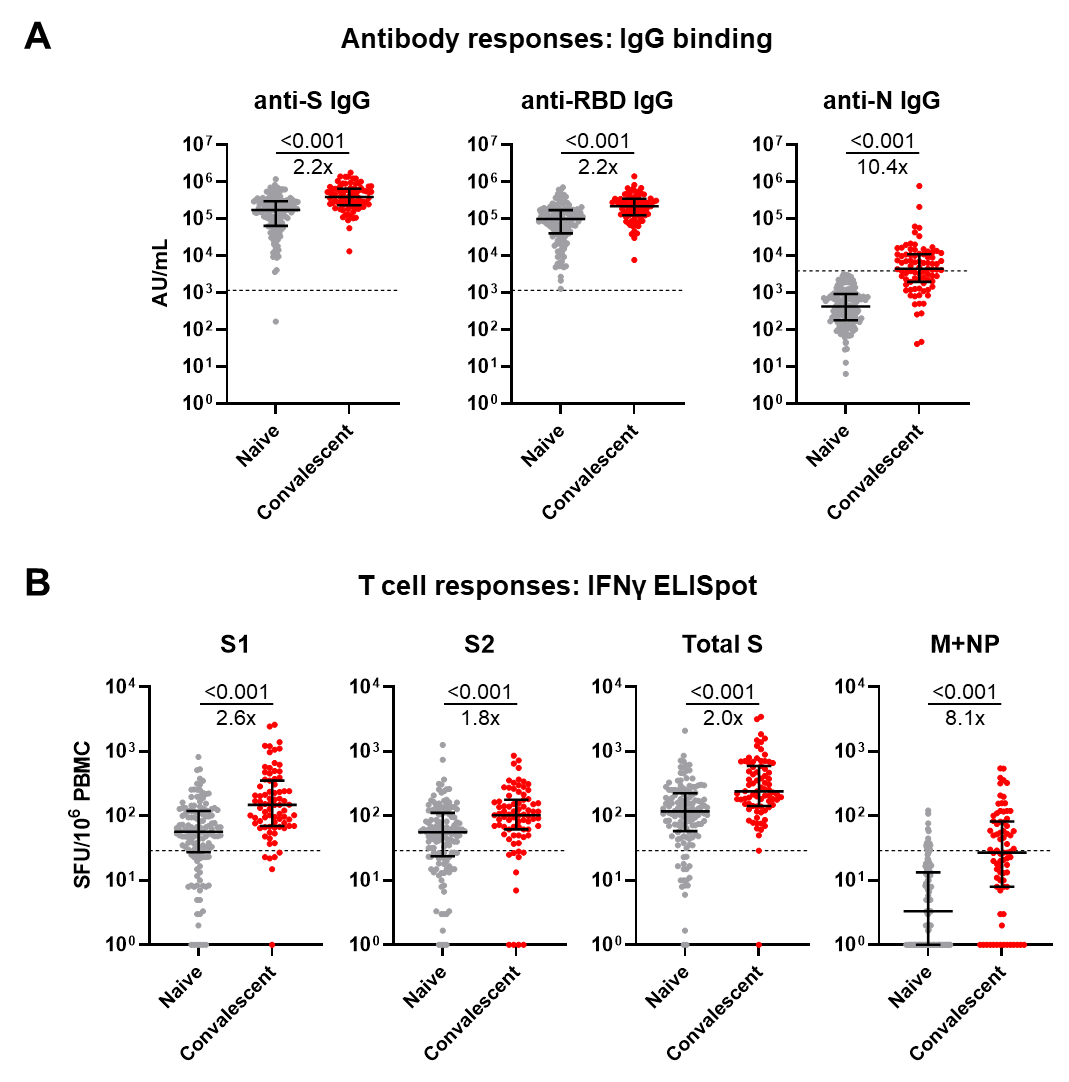
**Figure S2. Comparison of antibody and T cell responses between naive and previously infected individuals at 28 days after second vaccine dose. (A)** Ancestral SARS-CoV-2 spike- (S), receptor binding domain- (RBD) and nucleoprotein- (N) specific IgG binding titre in individuals selected for the case-control study according to infection history, as measured by MSD. Naïve represents those with no history of prior infection (n=149). Convalescent represents those with a history of infection prior to vaccination (n=83). Dashed lines represent threshold for positive response (SARS-CoV-2 S IgG 1160 AU/mL, RDB IgG 1169 AU/mL, N IgG 3874 AU/mL). **(B)** T cell responses to peptide pools representing ancestral SARS-CoV-2 S1, S2, total S (summation of S1 and S2 responses) and membrane (M) and nucleoprotein (N) responses in individuals selected for the case-control study according to infection history, as measured by IFNγ ELISpot assay. Naïve n=136, Convalescent n=79. Dashed lines represent threshold for positive response (29 SFU/10^6^ PBMC) calculated as mean background (DMSO) response + 2 standard deviations. Values of 0 are replaced with 1 for representation on the logarithmic scale. Grey circles represent naïve individuals, red circles represent convalescent individuals. Bars represent median of each group. Error bars represent interquartile range. Two-tailed p-values derived from Mann-Whitney U tests shown above linking lines. Fold change in median response shown below linking lines, calculated as median response for Convalescent divided by median response for Naïve.
