# Supplementary material for: CD4+ and CD8+ T cells and antibodies are associated with protection against Delta vaccine breakthrough infection: a nested case-control study within the PITCH study
Source: mBio. 2023 Sep 1;14(5):e01212-23. doi: 10.1128/mbio.01212-23 (PMC10653804; doi:10.1128/mbio.01212-23)
Supplement: Figure S3 — Additional T cell data. [file mbio.01212-23-s0003.docx]

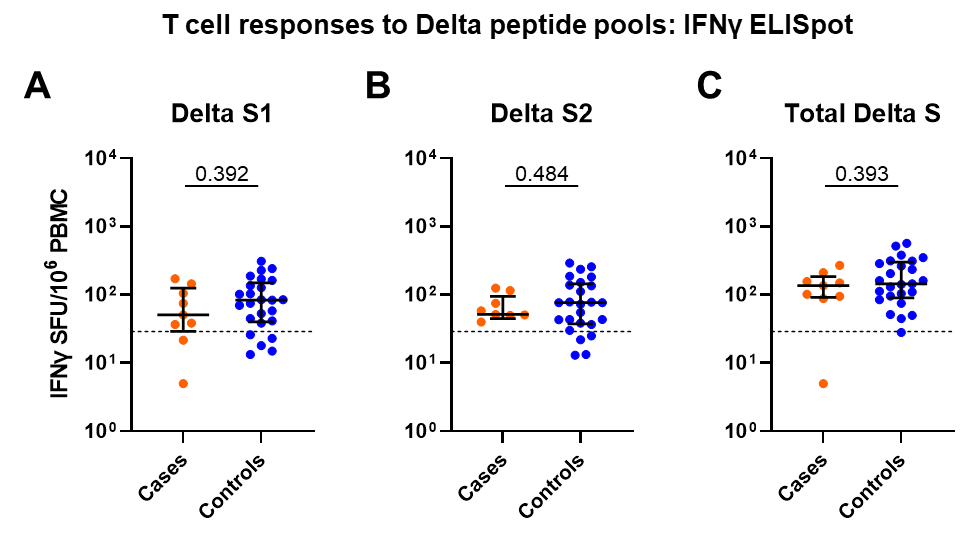
**Figure S3. Comparison of T cell responses to Delta spike peptide pools between cases and controls at 28 days after second vaccine dose.** T cell responses to peptide pools representing Delta (B1.617.2) SARS-CoV-2 **(A)** S1, **(B)** S2 and **(C)** Total Delta S (summation of Delta S1 and S2 responses) in a subset of cases (n=9) and controls (n=25), as measured by IFNγ ELISpot assay. Orange circles represent cases, blue circles represent controls. Bars represent median of each group. Error bars represent interquartile range. Two-tailed p-values derived from Mann-Whitney U tests shown above linking lines. Dashed lines represent threshold for positive response (29 SFU/10^6^ PBMC) calculated as mean background (DMSO) response + 2 standard deviations.
