# Supplementary material for: CD4+ and CD8+ T cells and antibodies are associated with protection against Delta vaccine breakthrough infection: a nested case-control study within the PITCH study
Source: mBio. 2023 Sep 1;14(5):e01212-23. doi: 10.1128/mbio.01212-23 (PMC10653804; doi:10.1128/mbio.01212-23)
Supplement: Figure S4 — Additional flow cytometry data. [file mbio.01212-23-s0004.docx]

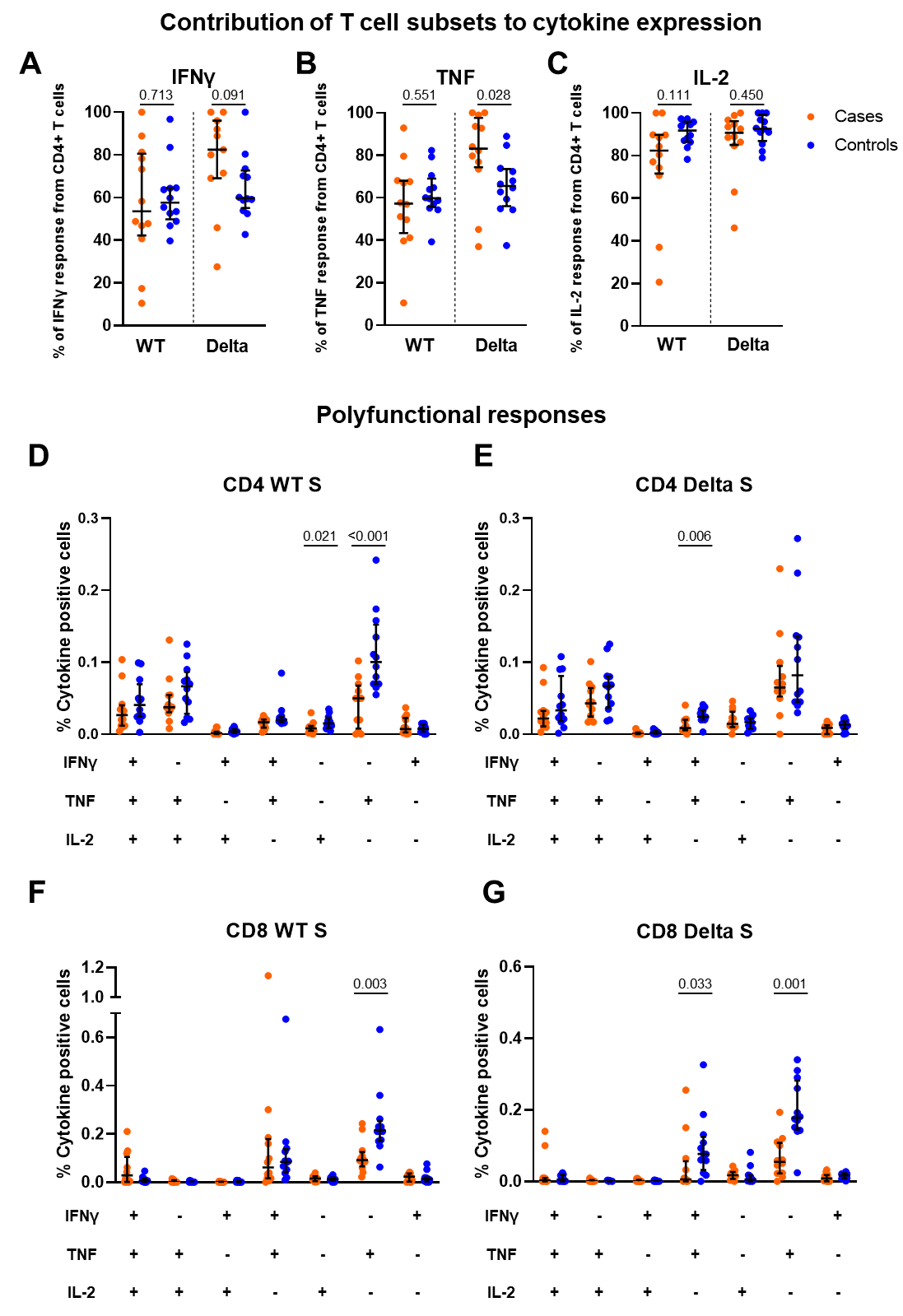
**Figure S4. Comparison of contribution of CD4+ T cell populations to cytokine responses and CD4+ and CD8+ T cell polyfunctionality between cases and matched controls at 28 days after second vaccine dose. (A)** T cell populations responsible for expression of IFNγ, **(B)** TNF and **(C)** IL-2, calculated by dividing the number of CD4+ cells expressing that cytokine after background subtraction, divided by the total number of CD4+ and CD8+ cells expressing that cytokine after background subtraction. **(D)** T cell polyfunctionality as assessed by combination of expression of IFNγ, IL-2 and TNF cytokines in CD4+ cells in response to ancestral (WT) spike, **(E)** CD4+ cells in response to Delta spike, **(F)** CD8+ cells in response to WT spike and **(G)** CD8+ cells in response to Delta spike peptide pools. Orange circles represent cases, blue circles represent controls. Bars represent median of each group. Error bars represent interquartile range. Two-tailed p-values derived from Mann-Whitney U tests shown above linking lines. For clarity, only p-values <0.05 shown in **(D-G).**
