# Supplementary material for: CD4+ and CD8+ T cells and antibodies are associated with protection against Delta vaccine breakthrough infection: a nested case-control study within the PITCH study
Source: mBio. 2023 Sep 1;14(5):e01212-23. doi: 10.1128/mbio.01212-23 (PMC10653804; doi:10.1128/mbio.01212-23)
Supplement: Figure S5 — Flow cytometry gating strategy. [file mbio.01212-23-s0005.docx]

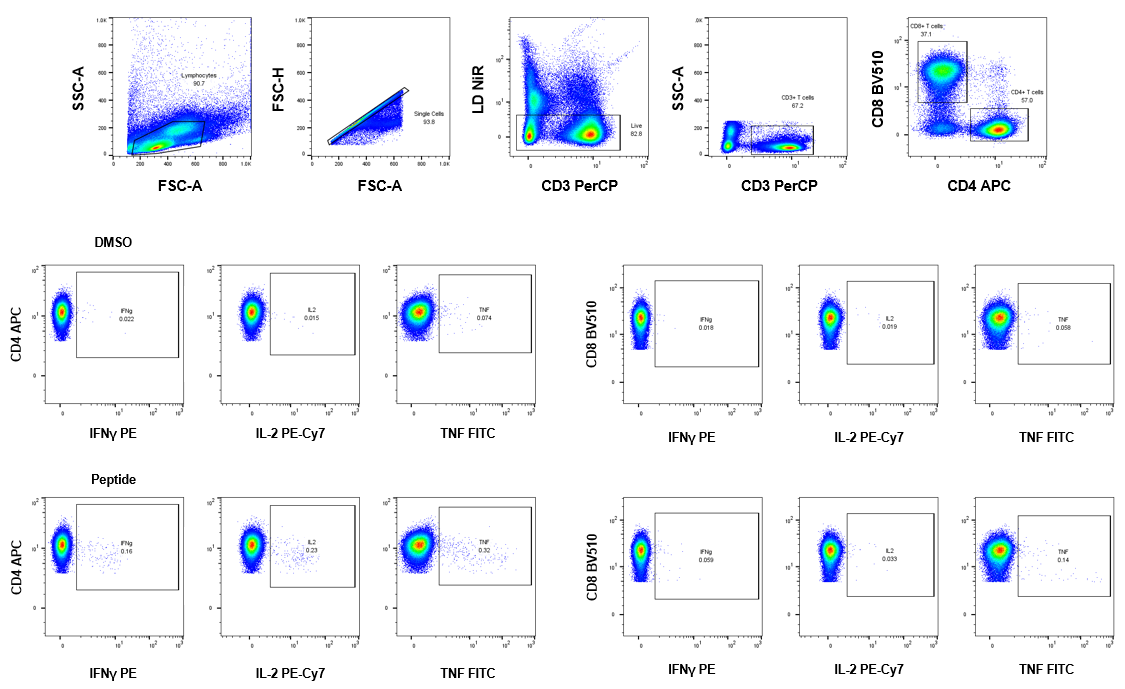


**Figure S5.** **Gating strategy for intracellular cytokine staining (ICS) assay.** Lymphocytes were gated using forward scatter (FSC)-area (A) and side scatter (SSC)-A. FSC-A and FSC-height (H) were then used to identify single cells. Live CD3+ T cells were gated by first excluding dead cells on the basis of exclusion of Live Dead stain (LD NiR) and then gating on positivity for CD3 PerCP. CD4+ and CD8+ subsets were identified based on staining for CD4 APC and CD8 BV510. Expression of IFNγ, IL-2 and TNF was identified in the CD4+CD8- and CD4-CD8+ gates. Representative gating shown for one sample for the negative control (DMSO) and ancestral S (peptide) stimulation conditions.
