# Supplementary material for: CD4+ and CD8+ T cells and antibodies are associated with protection against Delta vaccine breakthrough infection: a nested case-control study within the PITCH study
Source: mBio. 2023 Sep 1;14(5):e01212-23. doi: 10.1128/mbio.01212-23 (PMC10653804; doi:10.1128/mbio.01212-23)
Supplement: Secondary Author List — List of consortium authors. [file mbio.01212-23-s0006.docx]

**Secondary Author List**

Crick COVID Immunity Pipeline consortium authors, all affiliated with The Francis Crick Institute, London, UK:

Titilayo Abiola, Janet Abreu, Lorin Adams, Ana Agau-Doce, Karen Ambrose, Neil Bailey, Philip Bawumia, Annabel Borg, Mercedes Cabrera Jarana, Simon Caidan, Marie Caulfield, Bobbi Clayton, Ilenia D'Angelo, Giulia Dowgier, Steve Gamblin, Sonia Gandhi, Mike Gavrielides, Ruth Harvey, Lou Herman, Agnieszka Hobbs, Philip Hobson, Jag Kandasamy, Svend Kjaer, Murad Miah, Mauro Miranda, Sina Namjou, Nicola O'Reilly, Meghan Poulten, Martina Ragno, Mahbubur Rahman, Andrew Riddell, Chloë Roustan, Emma Russell, Samuel Sade, Sandeep Sandhar, Chelsea Sawyer, Vanessa Silva, Callie Smith, Amy Strange, Charles Swanton, Tom Taylor, Rachel Ulferts, Scott Warchal, Mary Y Wu.

PITCH Consortium authors:

| **Full Name** | **Institution** |
| --- | --- |
| Jenna Ablott | Sheffield Teaching Hospitals NHS Foundation Trust |
| Sandra Adele | University of Oxford |
| Zahra Ahmed | University of Birmingham |
| Saly Al-Taei | University of Birmingham |
| Ali Amini | University of Oxford |
| Adrienn Angyal | University of Sheffield |
| M. Azim Ansari | University of Oxford |
| Rachel Anslow | University of Oxford |
| Ana Atti | UK Health Security Agency |
| James Austin | University of Liverpool |
| Angela Bailey | Newcastle upon Tyne Hospitals NHS Foundation Trust |
| Natalie A. Barratt | University of Sheffield |
| Martin Bayley | University of Sheffield |
| Sagida Bibi | University of Oxford |
| Lucy H. Booth | University of Cambridge |
| Alice Bridges-Webb | University of Oxford |
| Rebecca Brown | University of Sheffield |
| Holly Caborn | Sheffield Teaching Hospitals NHS Foundation Trust |
| Jeremy Chalk | University of Oxford |
| Anu Chawla | Liverpool University Hospitals NHS Foundation Trust |
| Elizabeth Clutterbuck | University of Oxford |
| Christopher P. Conlon | University of Oxford |
| Andrew Cross | Liverpool University Hospitals NHS Foundation Trust |
| Debbie Cross | University of Oxford |
| Sophie Davies | University of Oxford |
| Catherine de Lara | University of Oxford |
| Wanwisa Dejnirattisai | University of Oxford |
| Christina Dold | University of Oxford |
| Thomas M. Drake | University of Edinburgh |
| Elena Efstathiou | University of Birmingham |
| David Eyre | University of Oxford |
| Alex Fairman | University of Sheffield |
| Sian Faustini | University of Birmingham |
| Andrew Filby | Newcastle University |
| Sarah Foulkes | UK Health Security Agency |
| John Frater | University of Oxford |
| Lisa Frending | University of Oxford |
| Oliver Galgut | University of Birmingham |
| Siobhan Gardiner | University of Oxford |
| Philip Goulder | University of Oxford |
| Jessica Gregory | Sheffield Teaching Hospitals NHS Foundation Trust |
| Irina Grouneva | University of Sheffield |
| Lotta Gustafsson | Sheffield Teaching Hospitals NHS Foundation Trust |
| Carl-Philipp Hackstein | University of Oxford |
| Callum Halstead | University of Oxford |
| Sophie Hambleton | Newcastle University |
| Muzlifah Haniffa | Newcastle University |
| Helen Hanson | Newcastle upon Tyne Hospitals NHS Foundation Trust |
| Alexander Hargreaves | University of Oxford |
| Kate Harrington | Sheffield Teaching Hospitals NHS Foundation Trust |
| Jenny Haworth | Newcastle upon Tyne Hospitals NHS Foundation Trust |
| Carole Hays | Newcastle upon Tyne Hospitals NHS Foundation Trust |
| Phoebe Hazenberg | Newcastle upon Tyne Hospitals NHS Foundation Trust |
| Luisa M. Hering | University of Liverpool |
| Emily C. Horner | University of Cambridge |
| Hailey Hornsby | University of Sheffield |
| Fatima Mariam Ilyas | Sheffield Teaching Hospitals NHS Foundation Trust |
| Jasmin Islam | UK Health Security Agency |
| Anni Jämsén | University of Oxford |
| Katie Jeffery | University of Oxford |
| Sile Johnson | University of Oxford |
| Geraldine Jones | Newcastle upon Tyne Hospitals NHS Foundation Trust |
| Mwila Kasanyinga | University of Oxford |
| Sinead Kelly | Newcastle upon Tyne Hospitals NHS Foundation Trust |
| Maqsood Khan | Sheffield Teaching Hospitals NHS Foundation Trust |
| Jon Kilby | University of Sheffield |
| Rosemary Kirk | Sheffield Teaching Hospitals NHS Foundation Trust |
| Allan Lawrie | University of Sheffield |
| Lauren Lett | University of Liverpool |
| Chang Liu | University of Oxford |
| Alison Lye | Sheffield Teaching Hospitals NHS Foundation Trust |
| Tom Malone | University of Oxford |
| Spyridoula Marinou | University of Oxford |
| Chloe Matthewman | Sheffield Teaching Hospitals NHS Foundation Trust |
| Philippa C. Matthews | Francis Crick Institute |
| David McDonald | Newcastle University |
| Jessica McNeill | Sheffield Teaching Hospitals NHS Foundation Trust |
| Gracie Mead | University of Oxford |
| Naomi Meardon | Sheffield Teaching Hospitals NHS Foundation Trust |
| Alexander J. Mentzer | University of Oxford |
| Shagun Misra | Sheffield Teaching Hospitals NHS Foundation Trust |
| Juthathip Mongkolsapaya | University of Oxford |
| Sam M. Murray | University of Oxford |
| Jeremy M. Nell | Newcastle upon Tyne Hospitals NHS Foundation Trust |
| Alexander R. Nicols | Newcastle University |
| Christopher Norman | Sheffield Teaching Hospitals NHS Foundation Trust |
| Ane Ogbe | University of Oxford |
| Juyeon Park | University of Oxford |
| Brendan A.I. Payne | Newcastle upon Tyne Hospitals NHS Foundation Trust |
| Eloise Phillips | University of Oxford |
| Gareth Platt | University of Liverpool |
| Andrew J. Pollard | University of Oxford |
| Sonia Poolan | Newcastle upon Tyne Hospitals NHS Foundation Trust |
| Nicholas Provine | University of Oxford |
| Chloe Roddis | Sheffield Teaching Hospitals NHS Foundation Trust |
| Stefan Roman | Sheffield Teaching Hospitals NHS Foundation Trust |
| Leigh Romaniuk | Newcastle upon Tyne Hospitals NHS Foundation Trust |
| Patpong Rongkard | University of Oxford |
| Sarah L. Rowland-Jones | University of Sheffield |
| Ayoub Saei | UK Health Security Agency |
| Jose Schutter | University of Sheffield |
| Gavin Screaton | University of Oxford |
| Adrian Shields | University of Birmingham |
| Laura Silva Reyes | University of Oxford |
| Donal Skelly | University of Oxford |
| Nikki Smith | University of Sheffield |
| Jarmila S. Spegarova | Newcastle University |
| Gareth Stephens | Sheffield Teaching Hospitals NHS Foundation Trust |
| Emily Stephenson | Newcastle University |
| Rachel Stimpson | Sheffield Teaching Hospitals NHS Foundation Trust |
| Scarlett Strickland | Sheffield Teaching Hospitals NHS Foundation Trust |
| Krishanthi Subramaniam | University of Liverpool |
| Piyada Supasa | University of Oxford |
| Chloe Tanner | University of Birmingham |
| Lydia J. Taylor | Newcastle University |
| Chitra Tejpal | University of Oxford |
| James E.D. Thaventhiran | University of Cambridge |
| Nicola Tinker | Sheffield Teaching Hospitals NHS Foundation Trust |
| Tom Tipton | University of Oxford |
| Neal Townsend | University of Birmingham |
| Simon Travis | University of Oxford |
| Nicola Trewick | Newcastle University |
| Stephanie Tucker | Newcastle University |
| Aekkachai Tuekprakhon | University of Oxford |
| Helena Turton | University of Sheffield |
| Jessica K. Tyerman | Newcastle University |
| Zara Valiji | University of Oxford |
| Lisa Watson | Sheffield Teaching Hospitals NHS Foundation Trust |
| Rachel Whitham | Sheffield Teaching Hospitals NHS Foundation Trust |
| Jayne Willson | Sheffield Teaching Hospitals NHS Foundation Trust |
| Barbara Wilson | Newcastle University |
| Joseph D. Wilson | University of Oxford |
| Steven Wood | University of Sheffield |
| Daniel G. Wootton | University of Liverpool |
| Amira A.T. Zawia | Sheffield Teaching Hospitals NHS Foundation Trust |
| Martha Zewdie | University of Oxford |
| Peijun Zhang | University of Sheffield |
