# Supplementary material for: CD4+ and CD8+ T cells and antibodies are associated with protection against Delta vaccine breakthrough infection: a nested case-control study within the PITCH study
Source: mBio. 2023 Sep 1;14(5):e01212-23. doi: 10.1128/mbio.01212-23 (PMC10653804; doi:10.1128/mbio.01212-23)
Supplement: Supplemental Tables — Tables S1-S5. [file mbio.01212-23-s0007.docx]

**SUPPLEMENTARY TABLES S1-S5**

**Table S1.** **Demographic characteristics of vaccine breakthrough cases compared to controls included in main correlates analysis.**

|  | **Cases (%)** | **Controls (%)** |
| --- | --- | --- |
| **Total N** | 32 | 247 |
| **Age (years)** |  |  |
| Median | 43 | 43 |
| Interquartile range | 31.5-49 | 34-53.5 |
| Range | 22-72 | 22-71 |
| **Sex** |  |  |
| Female | 26 (81.25%) | 180 (72.9%) |
| Male | 6 (18.75%) | 67 (27.1%) |
| **Ethnicity (self-reported)** |  |  |
| White | 26 (81.3%) | 175 (70.9%) |
| Asian | 1 (3.1%) | 20 (8.1%) |
| Other | 1 (3.1%) | 11 (4.5%) |
| Unreported | 4 (12.5%) | 41 (16.6%) |
| **BMI (kg/m^2^)** |  |  |
| Not obese (<30) | 11 (34.4%) | 98 (39.7%) |
| Obese (≥30) | 2 (6.3%) | 9 (3.6%) |
| Unreported | 19 (59.4%) | 140 (56.7%) |
| **Vaccine regimen** |  |  |
| AZD1222 | 9 (28.1%) | 32 (13.0%) |
| BNT162b2 (short interval^1^) | 2 (6.3%) | 58 (23.5%) |
| BNT162b2 (long interval^2^) | 21 (65.6%) | 157 (63.6%) |
| **Infection history** |  |  |
| Naïve | 26 (81.25) | 147 (59.5%) |
| Convalescent | 6 (18.75%) | 100 (40.5%) |

^1^Short interval represents 2-5 weeks between first and second dose.

^2^Long interval represents 6-14 weeks between first and second dose.

| **Immune parameter** | **Assay** | **Group** | **Number of individuals** | |
| --- | --- | --- | --- | --- |
|  |  |  | **Cases**  **(total N=32)** | **Controls**  **(total N=247)** |
| **IgG binding** | **Meso Scale Discovery assay (MSD)** | Total | **32** | **200** |
|  |  | AZ/Naive | 7 | 23 |
|  |  | AZ/Convalescent | 2 | 9 |
|  |  | Pfizer/Naive | 19 | 100 |
|  |  | Pfizer/Convalescent | 4 | 68 |
| **IFNγ response** | **T cell ELISpot (WT peptide pools)** | Total | **24** | **191** |
|  |  | AZ/Naive | 5 | 10 |
|  |  | AZ/Convalescent | 0 | 1 |
|  |  | Pfizer/Naive | 17 | 104 |
|  |  | Pfizer/Convalescent | 2 | 76 |
|  | **T cell ELISpot (Delta peptide pools)** | Total | **9** | **25** |
|  |  | AZ/Naive | 5 | 5 |
|  |  | AZ/Convalescent | 0 | 0 |
|  |  | Pfizer/Naive | 3 | 13 |
|  |  | Pfizer/Convalescent | 1 | 7 |
| **Neutralising antibody** | **Live virus microneutralisation assay** | Total | **21** | **41** |
|  |  | AZ/Naive | 3 | 7 |
|  |  | AZ/Convalescent | 1 | 0 |
|  |  | Pfizer/Naive | 14 | 19 |
|  |  | Pfizer/Convalescent | 3 | 15 |
| **B cell memory** | **B cell FluoroSpot** | Total | **10** | **11** |
|  |  | AZ/Naive | 2 | 2 |
|  |  | AZ/Convalescent | 0 | 0 |
|  |  | Pfizer/Naive | 8 | 9 |
|  |  | Pfizer/Convalescent | 0 | 0 |
| **CD4+ and CD8+ T cell response** | **Intracellular cytokine staining (ICS)** | Total | **12** | **12** |
|  |  | AZ/Naive | 2 | 2 |
|  |  | AZ/Convalescent | 0 | 0 |
|  |  | Pfizer/Naive | 10 | 10 |
|  |  | Pfizer/Convalescent | 0 | 0 |

**Table S2.** **Number and key characteristics of participants sampled for each assay.**

**Table S3.** **Demographic characteristics of vaccine breakthrough cases compared to matched controls included in B cell FluoroSpot and intracellular cytokine staining assays.**

|  | **Cases** | **Controls** |
| --- | --- | --- |
| **B cell FluoroSpot assay** | | |
| **Total N** | 10 | 11 |
| **Age (years)** |  |  |
| Median | 45 | 42 |
| Interquartile range | 39.8-49 | 40-51.5 |
| Range | 28-63 | 22-66 |
| **Sex** |  |  |
| Female | 9 | 9 |
| Male | 1 | 2 |
| **Vaccine regimen** |  |  |
| AZD1222 | 2 | 2 |
| BNT162b2 | 8 | 9 |
| **Intracellular cytokine staining (ICS) assay** | | |
| **Total N** | 12 | 12 |
| **Age (years)** |  |  |
| Median | 40.5 | 41.5 |
| Interquartile range | 31.8-51 | 30-47.8 |
| Range | 24-72 | 22-66 |
| **Sex** |  |  |
| Female | 11 | 11 |
| Male | 1 | 1 |
| **Vaccine regimen** |  |  |
| AZD1222 | 2 | 2 |
| BNT162b2 | 10 | 10 |

All individuals were infection-naïve before vaccination.

| **Marker name** | **Fluorochrome** | **Clone** | **Species reactivity** | **Host species** | **Isotype** | **Manufacturer** | **Catalogue number** | **Dilution used** |
| --- | --- | --- | --- | --- | --- | --- | --- | --- |
| CD3 | PerCP | UCHT1 | Human | Mouse | IgG1,κ | Biolegend | 300428 | 100 |
| CD4 | APC | RPA-T4 | Human | Mouse | IgG1,κ | Biolegend | 300514 | 200 |
| CD8 | BV510 | RPA-T8 | Human | Mouse | IgG1,κ | Biolegend | 301048 | 600 |
| CD14 | APC-Fire750 | M5E2 | Human | Mouse | IgG2a,κ | Biolegend | 301854 | 200 |
| IFNγ | PE | 4S.B3 | Human | Mouse | IgG1,κ | Biolegend | 502508 | 50 |
| TNF | FITC | MAb11 | Human | Mouse | IgG1,κ | Biolegend | 502906 | 40 |
| IL-2 | PE-Cy7 | MQ1-17H12 | Human | Rat | IgG2a,κ | eBioscience | 25-7029-41 | 100 |

**Table S4.** **Antibodies used for intracellular cytokine staining assay.**

**Table S5.** **Details of viruses used for live virus microneutralisation assay.**

| **Lineage** | **Mutations present in Spike** | **Source** |
| --- | --- | --- |
| Ancestral [hCoV19/England/02/2020] |  | Public Health England, UK |
| Delta  [MS066352H] | T19R, K77R, G142D, Δ156- 157/R158G, A222V, L452R, T478K, D614G, P681R, D950N | Prof. Wendy Barclay, Imperial College London, London, UK via the Genotype-to-Phenotype National Virology Consortium (G2P-UK) |
